# Postoperative hyper-inflammation as a predictor of poor outcomes in patients with acute type A aortic dissection (ATAAD) undergoing surgical repair

**DOI:** 10.1186/s13019-024-02637-7

**Published:** 2024-03-19

**Authors:** Yuan-Xi Luo, Yusanjan Matniyaz, Yu-Xian Tang, Yun-Xing Xue, Yi Jiang, Ke Pan, Zhi-Kang Lv, Zhi-Wei Fan, Kuo Wang, Hai-Tao Zhang, He Zhang, Wen-Zhe Wang, Tuo Pan, Dong-Jin Wang, Fu-Dong Fan

**Affiliations:** 1 Department of Cardio-Thoracic Surgery, Nanjing Drum Tower Hospital, Chinese Academy of Medical Sciences & Peking Union Medical College, Nanjing, China; 2 Department of Cardio-Thoracic Surgery, Nanjing Drum Tower Hospital, affiliated Hospital of Medical School, Nanjing University, Number 321 Zhongshan Road, Nanjing, 210008 Jiangsu China; 3 Department of Cardio-Thoracic Surgery, Nanjing Drum Tower Hospital Clinical College of Nanjing University of Chinese Medicine, Nanjing, China; 4 Department of Cardio-Thoracic Surgery, Nanjing Drum Tower Hospital Clinical College of Xuzhou Medical University, Nanjing, China; 5 Department of Cardio-Thoracic Surgery, Affiliated Drum Tower Hospital, Medical School of Nanjing University, Nanjing, 210008 China

**Keywords:** Hyper-inflammation, Acute Stanford type A aortic dissection, Cardiopulmonary bypass, Deep hypothermia circulatory arrest, Mortality, Morbidity

## Abstract

**Background:**

Postoperative hyper-inflammation is a frequent event in patients with acute Stanford type A aortic dissection (ATAAD) after surgical repair. This study's objective was to determine which inflammatory biomarkers could be used to make a better formula for identifying postoperative hyper-inflammation, and which risk factors were associated with hyper-inflammation.

**Methods:**

A total of 405 patients were enrolled in this study from October 1, 2020 to April 1, 2023. Of these patients, 124 exhibited poor outcomes. In order to investigate the optimal cut-off values for poor outcomes, logistic and receiver operating characteristic analyses were performed on the following parameters on the first postoperative day: procalcitonin (PCT), C-reactive protein (CRP), interleukin-6 (IL-6), and systemic immune-inflammation index (SII). These cut-off points were used to separate the patients into hyper-inflammatory (n = 52) and control (n = 353) groups. Finally, the logistic were used to find the risk factors of hyper-inflammatory.

**Results:**

PCT, CRP, IL-6, and SII were independent risk factors of poor outcomes in the multivariate logistic model. Cut-off points of these biomarkers were 2.18 ng/ml, 49.76 mg/L, 301.88 pg/ml, 2509.96 × 10^9^/L respectively. These points were used to define postoperative hyper-inflammation (OR 2.97, 95% CI 1.35–6.53, *P* < 0.01). Cardiopulmonary bypass (CPB) > 180 min, and deep hypothermia circulatory arrest (DHCA) > 40 min were the independent risk factors for hyper-inflammation.

**Conclusions:**

PCT > 2.18, CRP > 49.76, IL-6 > 301.88, and SII < 2509.96 could be used to define postoperative hyper-inflammation which increased mortality and morbidity in patients after ATAAD surgery. Based on these findings, we found that CPB > 180 min and DHCA > 40 min were separate risk factors for postoperative hyper-inflammation.

## Introduction

Acute type-A aortic dissection (ATAAD) is a life-threatening disease that carries a high mortality risk. According to the findings of the international Registry of Acute Aortic Dissection (IRAD), the mortality rate among patients who underwent surgical repair ranged from 18 to 25%. In a word, despite the significant advancements in peri-operative strategies and surgical techniques over the past two decades, ATAAD remains a highly severe aortic condition [1]. Prolonged cardiopulmonary bypass (CPB), aortic cross-clamp (ACC) and deep hypothermia circulatory arrest (DHCA) were ordinary during ATAAD surgical repair, which lead to various components of the inflammatory response were documented [2–7]. Consequently, patients with ATAAD who underwent surgical repair often experienced postoperative hyper-inflammatory conditions compared with other common cardiac surgeries, such as myocardial ischemia–reperfusion injury, coagulopathy, hypotension or shock postoperative and acute lung injury or even acute respiratory distress syndrome (ARDS) [1, 8–10].

Over the past years, numerous clinical studies have been conducted to identify risk factors associated with poor outcomes in order to enable early diagnosis and treatment of ATAAD, which included hyper-inflammatory complications [8, 10, 11]. During CPB in ATAAD surgery, an amplified systemic inflammatory response, involving neutrophile, lymphocyte, platelet, C reactive protein (CRP), procalcitonin (PCT), and interleukin-6 (IL-6) in body, was demonstrated that had a significant association with unfavorable postoperative outcomes including coagulopathy, surgical bleeding, re-intubation and so on [2–5, 12]. It has been proven that an early-initiated anti-inflammatory strategy is a promising option after ATAAD surgery [6]. Therefore, early prediction of hyper-inflammation after ATAAD may be useful and valuable to decrease mortality and morbidity.

There was a large body of research investigating association between postoperative hyper-inflammation and adverse outcomes after ATAAD surgery [8, 10, 11]. Li et al. [11] conducted a study where they found that the systemic immune-inflammation index (SII), a reliable indicator of systemic inflammation based on peripheral lymphocyte, neutrophil, and platelet, could effectively predict the survival of patients following surgery. Their methodology interested us deeply in investigating the definition of postoperative hyper-inflammation. However, it may be biased to make a statistical model on an isolated variable due to interference of per-operative and surgical strategies, such as CPB, ACC, DHCA and so on. Therefore, we designed this retrospective cohort study to determine which inflammatory biomarkers could be used to make a better formula for identifying postoperative hyper-inflammation, and which risk factors were associated with hyper-inflammation.

## Methods

### Patients

This study received approval from the ethical committees of Nanjing Drum Tower Hospital (No.2020-185-01). It was not feasible to obtain informed consent from all patients due to the nature of the study. However, since this study posed no risk to the patients involved, the institutional ethics committee waived the requirement for informed consent. The study adhered strictly to the Declaration of Helsinki (seventh revision, 2013) and was conducted under the supervision of the ethics committee. After obtaining approval from the ethical committees, a review was conducted using hospital medical records, nursing records, laboratory data, and surgical databases at a tertiary hospital.

*Inclusion criteria* Adult patients (aged 18 years old and above) who underwent surgery for ATAAD at our center would be enrolled in this study. *Exclusion criteria* Patients would be excluded when they met any of the following criteria: experienced a cardiogenic shock at the conclusion of CPB (vasoactive inotropic score > 40, cardiac index < 2.2 L/min m^2^, mean arterial pressure < 65 mm Hg)[7]; diagnosis of inflammatory immune diseases, infectious diseases, or tumor diseases; previous treatment with immune-suppressing medications; initiation of extracorporeal membrane oxygenation (ECMO) or continuous renal replacement therapy (CRRT) before surgery; had mechanically ventilated before surgery; received DAVID procedure; pregnancy.

Primary outcome of this study was a composite of unfavorable morbidity following surgery that called “the poor outcome”. Morbidity was considered present if any of the following postoperative conditions were observed: in-hospital death, ECMO or CRRT use, tracheal re-intubation, severe pneumonia, mechanical ventilation time > 72 h, and malperfusion syndrome. Secondary endpoints included preoperative and postoperative inflammatory indicators such as SII, CRP, PCT and IL-6; observed surgical duration (CPB, ACC, DHCA runs).

### Surgical intervention

Upon diagnosis of ATAAD, patients were promptly transferred to the intensive care unit. Central repair surgery was performed within 24 h of symptom onset. Patients experiencing critical conditions such as cardiac tamponade, cardiogenic shock, cerebrovascular accident, stroke, coma, myocardial ischemia, acute renal failure, or mesenteric ischemia would experience emergency surgery. The surgical approach involved a standard median sternotomy, with cannulation of the femoral artery, right axillary artery, and right atrium for CPB circulation. Antegrade cerebral perfusion was ensured via cannulation of the right axillary artery. Systemic temperature was maintained between 32 and 34 °C, and CPB was arrested once the desired hypothermic circulatory arrest temperature (22–24 °C) was reached. The initial volume of the antegrade cold blood cardioplegia solution, in a 4:1 ratio, was administered to achieve complete cessation of all cardiac electrical activity, with a minimum requirement of 20 ml/kg. To maintain cardiac arrest, retrograde infusion of 10 ml/kg of blood cardioplegia solution was repeated every 15 min after 30 min of initial antegrade infusion. If retrograde infusion was used three times, the antegrade strategy would be implemented accordingly.

The choice of distal surgical method depends on the location of the intimal tear and the extent of dissection. In cases where the primary tear is in the ascending aorta and the dissection is limited (arch dilation < 50 mm) [13], an ascending aortic replacement + hemi-arch replacement without antegrade stent-implantation is feasible. Otherwise, alternatives include ascending aortic replacement + total arch replacement + a frozen elephant trunk (FET) (MicroPort Medical Co Ltd) or ascending aortic replacement + an arched fenestrated stent graft (FSG) (Yuhengjia Sci-Tech Corp Ltd) implantation intraoperatively [14, 15]. For proximal segments, root reinforcement reconstruction or double jacket wrapping [16] were routinely performed. In situations where the dissection involves the coronary ostia, aortic valve, and aortic root aneurysm, the Bentall procedure and Wheat's procedure would be employed. If the dissection impairs the coronary artery, coronary artery bypass grafting (CABG) would be performed.

### Data collection and definition

Baseline characteristics, laboratory features, operative details, and outcome data were collected from our electronic medical record database. The biomarkers of inflammation, including Neutrophile, Lymphocyte, Platelet, CRP, PCT, as well as IL-6 levels on the first postoperative day (POD1) were also collected. The formula of SII is as follows: platelet count × neutrophil to lymphocyte ratio [11]. Pre-operative ischemia (Table 1) could be considered if met the following condition: the presence of impaired blood flow in brain, coronary artery, limb and bowel as seen on radiographs; significant clinical symptoms, such as changes of pupillary size and light reaction pain, vomiting, bloody stool, abdominal pain, pallor, paresthesia, poikilothermia, or paralysis, etc. Malperfusion syndrome was defined as the presence of any organ malperfusion prior to surgery. According to our previous report, it includes cardiac malperfusion, cerebral malperfusion, renal malperfusion, mesenteric malperfusion, lower limb malperfusion [10].

**Table 1:** Baseline characteristics

Variables	Control (n = 281)	Poor outcome (n = 124)	*P* value
Age(year)	53.8 ± 13.4	55.3 ± 13.5	0.371
Gender male n (%)	217 (77.2)	93 (75.0)	0.626
BMI(kg/m^2^)	25.4 (23.3–28.4)	25.2 (20.9–27.7)	0.570
*Medical history n (%)*			
Hypertension	271 (96.4)	116 (93.5)	0.193
Diabetes mellitus	50 (17.8)	30 (24.2)	0.136
COPD	98 (34.9)	45 (36.3)	0.784
Smoking	135 (48.0)	58 (46.8)	0.814
Heavy alcohol drinking	41 (14.6)	17 (13.7)	0.816
Liver diseases	12 (4.3)	1 (0.8)	0.068
Marfan syndrome	14 (5.0)	6 (4.8)	0.951
Cerebral infarction	12 (4.3)	5 (4.0)	0.912
Chronic renal failure	3 (1.1)	1 (0.8)	0.806
Ascending aortic diameter (cm)	4.7 (4.4–5.4)	5.1 (4.5–5.5)	0.003
Pre-operative PCT (ng/ml)	0.01 (0.01–0.1)	0.01 (0.01–0.1)	0.207
Pre-operative CRP (mg/L)	3.52 (1.14–7.8)	3.6 (1.5–6.0)	0.984
Pre-operative IL-6 (pg/ml)	34.7 (11.9–60.0)	40 (17.4–61.6)	0.205
Pre-operative SII	546 (153–1326)	539 (152–1512)	0.874
Pre-operative Neutrophile (× 10^9^/L)	4.3 (1–9.2)	9.2 (4.8–13.0)	0.822
Pre-operative Lymphocyte (× 10^9^/L)	1.0 (0.7–1.4)	0.9 (0.8–1.3)	0.556
Pre-operative Platelet (× 10^9^/L)	155 (116–190)	153 (121–176)	0.776
*Preoperative conditions n (%)*			
Bowel ischemia	0	7 (5.6)	< 0.001
Cerebral ischemia	0	42 (10.4)	< 0.001
Limb ischemia	0	51 (41.1)	< 0.001
Coronary artery involvement	7 (2.5)	6 (4.8)	0.217
*Arch surgery n (%)*			0.702
Total arch replacement	183 (65.2)	86 (69.4)	
Hemi arch replacement	40 (14.2)	16 (12.9)	
Arch stent	58 (20.6)	22 (17.7)	
*Root surgery n (%)*			0.304
Bentall procedure	144 (51.2)	65 (52.4)	
Wheat’s procedure	56 (25.7)	36 (29.0)	
Reconstruction	81 (23.1)	23 (18.6)	
Concomitant CABG n (%)	7 (2.5)	6 (4.8)	0.217
CPB time (min)	182 (158.5–220.5)	188 (163–228)	0.233
ACC time (min)	133 (110.5–169.0)	135 (120–175)	0.099
DHCA time (min)	33 (26–41)	31 (25–43)	0.594

Median (Interquartile range); *BMI* Body mass index, *COPD* Chronic obstructive pulmonary disease, *PCT* Procalcitonin, *CRP* C-reactive protein, *IL-6* interleukin-6, *SII* Systemic immune-inflammation index, *CABG* Coronary artery bypass graft, *CPB* Cardiopulmonary bypass, *ACC* Aortic cross-clamp, *DHCA* Deep hypothermia circulatory arrest

### Statistical analysis

Statistical analysis was conducted using IBM SPSS Statistics for Windows (version 24, IBM Corporation, Armonk, NY, USA). Continuous variables were generally described as mean ± SD or median with interquartile ranges (IQR), while categorical variables were expressed as frequencies (n, %). The Student t-test was utilized for normally distributed continuous variables, while the Mann–Whitney U nonparametric method was employed for non-normally distributed continuous variables. Categorical data were compared using either the chi-square test or Fisher exact test. The receiver operating characteristic (ROC) curve and Youden index were employed to assess the predictive values and cut-off points of SII, PCT, CRP and IL-6 on POD1 for poor outcomes, which were used to defined hyper-inflammation. The areas under the ROC curves (AUC) were used to determine predictive accuracy. Logistic regression analyses were performed to identify independent risk factors for poor outcomes and hyper-inflammation. Covariates reaching statistical significance (*P* ≤ 0.10) in the univariate analysis and those considered clinically relevant were entered into a multivariable logistic regression model. Collinearity and calibration were assessed for each multivariable logistic model using the variance inflation factor (VIF) and Hosmer–Lemeshow test, respectively. A two-sided *P*-value of < 0.05 was considered statistically significant.

## Results

A total of 426 patients underwent surgery for ATAAD at our hospital between October 1, 2020, and April 1, 2023. Among these patients, 405 individuals met the specified inclusion and exclusion criteria, and 124 exhibited poor outcomes which included in-hospital death (n = 43), ECMO (n = 5), tracheal re-intubation (n = 9), mechanical ventilation > 72 h (n = 54), severe pneumonia (n = 9), malperfusion syndrome (n = 97), and CRRT (n = 45). The detailed demographic and baseline data were provided in Table 1. Compared with the control, poor outcome group had higher level of ascending aortic diameter (median: 4.7 cm, IQR 4.4–5.4 cm vs. Median: 5.1 cm, IQR 4.5–5.5 cm, *P* = 0.003), and higher rate of pre-operative bowel ischemia (0 vs. 5.6%, *P* < 0.001), cerebral ischemia (0 vs. 10.4%, *P* < 0.001) and limb ischemia (0 vs. 41.1%, *P* < 0.001). Comparison of postoperative data revealed that poor outcome group had higher level of PCT (*P* < 0.001), CRP (*P* < 0.001) and IL-6 (*P* < 0.001), and lower level of SII (*P* < 0.001) compared with control group.

In multivariate analysis (Table 3), PCT, CRP, IL-6 and SII were independent risk factors of poor outcomes. Therefore, the PCT, CRP, IL-6 and SII were put into the ROC model. The cut-off points of these variables were 2.18 ng/ml (AUC: 0.61, sensitivity: 0.86, specificity: 0.38), 49.76 mg/L (AUC: 0.61, sensitivity: 0.85, specificity: 0.32), 301.88 pg/ml (AUC: 0.64, sensitivity: 0.44, specificity: 0.81), and 2509.96 × 10^9^/L (AUC: 0.69, sensitivity: 0.58, specificity: 0.76), respectively. Then, these cut-off points were used to define hyper-inflammation which increased the risk of poor outcome (Table 2: 5.3% vs. 29.8%, *P* < 0.001). After collinearity analysis, the multivariable regression model (Table 3) also showed that hyper-inflammation was an independent risk factor of poor outcomes (OR 2.97, 95% CI 1.35–6.53, *P* < 0.01). However, after regression adjustment, liver diseases, ascending aortic diameter and ACC time did not display a significant correlation with the poor outcomes.

**Table 2:** Postoperative outcomes

Variables	Control (n = 281)	Poor outcome (n = 124)	*P* value
Poor outcomes			
In-hospital death n (%)	–	43 (34.7)	–
ECMO n (%)	–	5 (4.0)	–
Re-intubation n (%)	–	9 (7.3)	–
MV > 72 h n (%)	–	54 (43.5)	–
Sever pneumonia n (%)	–	9 (7.3)	–
CRRT n (%)	–	45 (36.3)	–
Malperfusion syndrome n (%)	–	97 (78.2)	–
PCT on POD1 (ng/ml)	3.04 (2.03–5.6)	4.07 (2.6–6.6)	< 0.001
CRP on POD1 (mg/L)	77 (43.0–106.2)	99 (66–125)	< 0.001
IL-6 on POD1 (pg/ml)	154.7 (54.6–270.7)	239.3 (115.3–438.9)	< 0.001
SII on POD1	2963.9 (1634.5–5220.4)	1608.8 (876.9–2495.5)	< 0.001
Neutrophile on POD1 (× 10^9^/L)	9.1 (6.2–10.6)	9.15 (6.2–11.0)	0.422
Lymphocyte on POD1 (× 10^9^/L)	0.3 (0.2–0.3)	0.5 (0.3–0.7)	< 0.001
Platelet on POD1 (× 10^9^/L)	102 (76–125)	94 (68–123)	0.071
Hyper-inflammation n (%)	15 (5.3)	37 (29.8)	< 0.001

Median (Interquartile range); *ECMO* Extracorporeal membrane oxygenation, *MV* Mechanical ventilation, *CRRT* Continuous renal replacement therapy, *PCT* Procalcitonin, *CRP* C-reactive protein, *IL-6* Interleukin-6, *SII* Systemic immune-inflammation index, *POD1* The first postoperative day

**Table 3:** Multivariable logistic regression for poor outcomes

Variables	Odds ratio	95% CI	*P* value
Liver diseases	0.21	0.03–1.71	0.177
Ascending aortic diameter	1.01	1.00–1.01	0.068
ACC time	1.00	0.97–1.03	0.273
PCT on POD1	1.50	1.03–2.17	0.035
CRP on POD1	1.01	1.00–1.01	0.027
IL-6 on POD1	1.00	1.00–1.01	0.042
SII on POD1	1.00	1.00–1.01	0.002
Hyper-inflammation	2.97	1.35–6.53	0.007

*CI* Confidence interval, *ACC* Aortic cross-clamp, *POD1* The first postoperative day, *PCT* procalcitonin, *CRP* C-reactive protein, *IL-6* Interleukin-6, *SII* Systemic immune-inflammation index

**Fig. 1:**
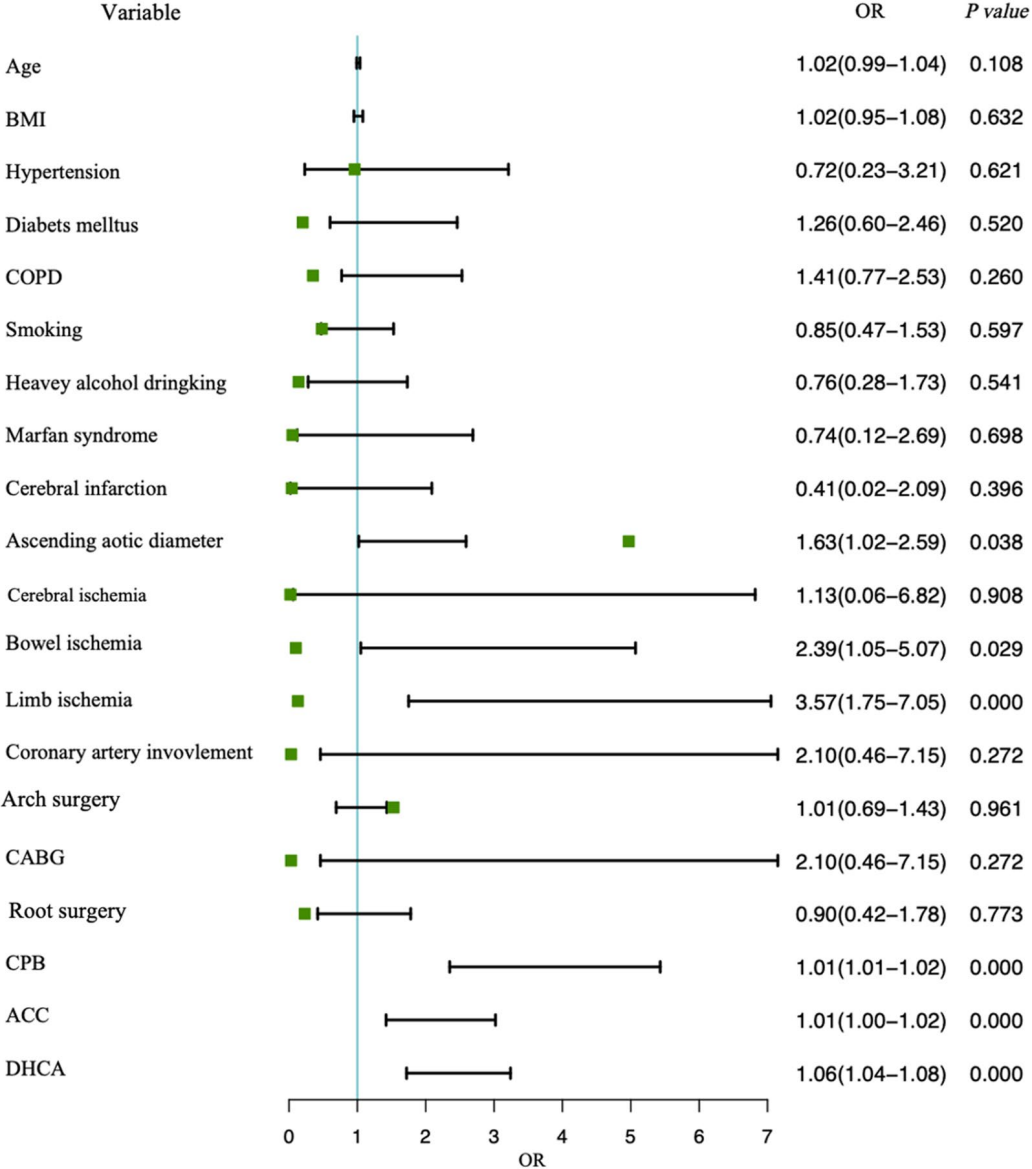
Univariable analysis for postoperative hyper-inflammation in patients with AAAD

**Fig. 2:**
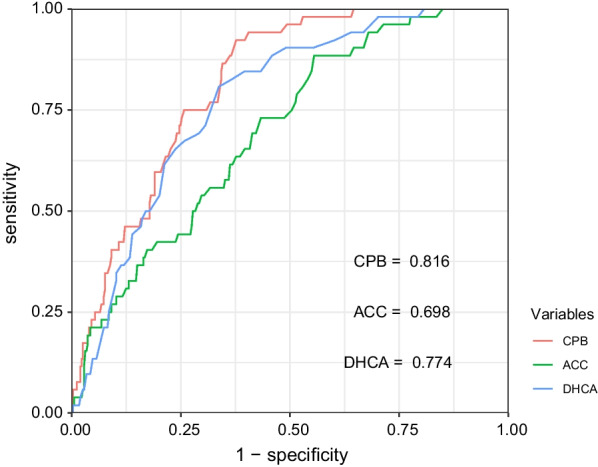
Receiver operating characteristic curve (ROC) analyses with the area under the curve, sensitivity and specificity of CPB, ACC, DHCA in predicting postoperative hyper-inflammation

Subsequently, the study cohort was divided into two groups: the hyper-inflammation group (n = 52) and the control group (n = 353) (Table 4). Contrast to the control group, the hyper-inflammatory group demonstrated a greater proportion of preoperative cerebral ischemia [10 (19.23) vs. 32 (9.07), *p* = 0.025] and limb ischemia [15 (28.85) vs. 36 (10.20), *p* < 0.001]. In the hyper-inflammatory group, patients tended to suffer longer CPB [218.00 (195.50–269.00) vs. 163.00 (140.00–196.00), *p* < 0.001], ACC [158.50 (133.75–207.50) vs. 134.00 (113.00–168.00), *p* < 0.001] and DHCA duration [46.00 (38.00–56.25) vs. 32.00 (25.00–41.00), *p* < 0.001]. Consistently as before, inflammatory fators on the first postoperative day were also significantly elevated in the high inflammation group. Notably, mortality was significantly higher in the population of hyper-inflammation group [16 (30.77) vs. 27 (7.65)]. In univariable analysis (Fig. 1), ascending aortic diameter, pre-operative bowel ischemia, pre-operative limb ischemia, CPB, ACC, and DHCA were associated with an increased incidence of postoperative hyper-inflammation. In the ROC model (Fig. 2), cut-off points of CPB, ACC and DHCA were 176.6≈180 min (AUC: 0.82, sensitivity: 0.88, specificity: 0.63), 127.5 min≈130 min (AUC: 0.70, sensitivity: 0.88, specificity: 0.44), and 37.7≈40 min (AUC: 0.77, sensitivity: 0.81, specificity: 0.66), respectively. In Table 5, the further multivariate regression model showed that pre-operative bowel ischemia (*P* = 0.004), pre-operative limb ischemia (*P* = 0.001), CPB > 180 min (*P* < 0.001), and DHCA > 40 min (*P* < 0.001) were independent risk factor of postoperative hyper-inflammation. The ACC > 130 min could not significantly increase the rate of postoperative hyper-inflammation (*P* = 0.397).

**Table 4:** Comparison of population with and without postoperative hyper-inflammation

Variables	Control (n = 353)	Hyper-inflammation (n = 52)	*P* value
Age(year)	53.84 ± 13.59	57.06 ± 12.30	0.107
Gender male n (%)	273 (77.34)	37 (71.15)	0.326
BMI(kg/m^2^)	25.37 (23.32–27.91)	24.83 (22.20–28.53)	0.769
*Medical history n (%)*			
Hypertension	387 (95.56)	338 (95.75)	0.892
Diabetes mellitus	68 (19.26)	12 (23.08)	0.519
COPD	121 (34.28)	22 (42.31)	0.258
Smoking	170 (48.16)	23 (44.23)	0.596
Heavy alcohol drinking	52 (14.73)	6 (11.54)	0.540
Liver diseases	13 (3.68)	0 (0.00)	0.325
Marfan syndrome	18 (5.10)	2 (3.85)	0.963
Cerebral infarction	16 (4.53)	1 (1.92)	0.613
Chronic renal failure	4 (1.13)	0 (0.00)	1.000
Ascending aortic diameter (cm)	4.80 (4.40–5.50)	4.90 (4.50–5.53)	0.020
Pre-operative PCT (ng/ml)	0.01 (0.01–0.10)	0.01 (0.01–0.10)	0.877
Pre-operative CRP (mg/L)	3.52 (1.14–7.77)	3.92 (1.14–16.10)	0.596
Pre-operative IL-6 (pg/ml)	34.70 (11.68–52.71)	39.85 (17.40–107.75)	0.063
Pre-operative SII	531.84 (153.00–1400.00)	615.55 (184.46–1400.00)	0.669
*Preoperative conditions n (%)*			
Bowel ischemia	6 (1.70)	1 (1.92)	1.000
Cerebral ischemia	32 (9.07)	10 (19.23)	0.025
Limb ischemia	36 (10.20)	15 (28.85)	< 0.001
Coronary artery involvement	10 (2.83)	3 (5.77)	0.484
*Arch surgery n (%)*			0.941
Total arch replacement	235 (66.57)	34 (65.38)	
Hemi arch replacement	48 (13.60)	8 (15.38)	
Arch stent	70 (19.83)	10 (19.23)	
*Root surgery n (%)*			0.461
Bentall procedure	185 (52.41)	24 (46.15)	
Wheat’s procedure	81 (22.95)	11 (21.15)	
Reconstruction	87 (24.65)	17 (32.69)	
Concomitant CABG n (%)	10 (2.83)	3 (5.77)	0.484
CPB time (min)	163.00 (140.00–196.00)	218.00 (195.50–269.00)	< 0.001
ACC time (min)	134.00 (113.00–168.00)	158.50 (133.75–207.50)	< 0.001
DHCA time (min)	32.00 (25.00–41.00)	46.00 (38.00–56.25)	< 0.001
PCT on POD1 (ng/ml)	3.04 (2.03–5.48)	6.34 (3.57–6.90)	< 0.001
CRP on POD1 (mg/L)	79.00 (44.00–113.00)	104.00 (69.00–125.50)	< 0.001
IL-6 on POD1 (pg/ml)	154.70 (54.62–253.52)	438.86 (329.28–1039.78)	< 0.001
SII on POD1	2698.75 (1516.67–4891.25)	1259.13 (714.77–1692.67)	< 0.001
Death	27 (7.65)	16 (30.77)	< 0.001

Median (Interquartile range); *BMI* Body mass index, *COPD* Chronic obstructive pulmonary disease, *PCT* Procalcitonin, *CRP* C-reactive protein, *IL-6* Interleukin-6, *SII* Systemic immune-inflammation index, *CABG* Coronary artery bypass graft, *CPB* Cardiopulmonary bypass, *ACC* Aortic cross-clamp, *DHCA* Deep hypothermia circulatory arrest

**Table 5:** Multivariable logistic regression for postoperative hyper-inflammation

Variables	Odds ratio	95% CI	*P* value
Ascending aortic diameter > 5 cm [20]	1.14	0.58–2.26	0.703
Pre-operative bowel ischemia	4.19	1.59–11.03	0.004
Pre-operative limb ischemia	4.07	1.78–9.29	0.001
Cardiopulmonary bypass time > 180 min	14.16	4.10–48.82	< 0.001
Aortic cross-clamping time > 130 min	0.61	0.19–1.92	0.397
DHCA time > 40 min	4.11	2.03–8.31	< 0.001

*CI* Confidence interval, *CPB* Cardiopulmonary bypass, *ACC* Aortic cross-clamp, *DHCA* Deep hypothermia circulatory arrest

## Discussion

The hyper-inflammatory diseases, which usually result in poor prognosis, are common complications in patients after ATAAD surgery [8, 10, 11]. However, it had no large-sample-sized study to demonstrate the identification of postoperative hyper-inflammation in patients with ATAAD. Based on our center's cohort of ATAAD patients October 1, 2020 to April 1, 2023, we found that ① an easy formula could be used to define postoperative hyper-inflammation (PCT > 2.18 ng/ml & CRP > 49.76 mg/L & IL-6 > 301.88 pg/ml & SII < 2509.96 × 10^9^/L); ② The morbidity of hyper-inflammation was about 12.83% in patients after ATAAD surgery, which was significantly associated with high mortality and morbidity; ③ the pre-operative bowel ischemia, pre-operative limb ischemia, CPB > 180 min and DHCA > 40 min were independent risk factors for postoperative hyper-inflammation.

To our best known, postoperative hyper-inflammatory diseases in patients with ATAAD include acute lung injury, malperfusion syndrome, and severe pneumonia [8, 10, 11]. The systemic inflammatory response could induce acute lung injury which could significantly increase in-hospital death [6, 17]. The malperfusion syndrome which is actually an ischemia–reperfusion injury (I/R injury), is caused by an immune-inflammatory response, which has been proven the lethal factor of in-hospital death [1, 10]. Severe pneumonia which is significantly associated with poor outcome, result in systemic inflammatory response syndrome (SIRS) or septic shock [18]. We, therefore, defined poor outcomes based on these poor inflammation-related endpoints, including in-hospital death, ECMO, tracheal re-intubation, mechanical ventilation > 72 h, severe pneumonia, malperfusion syndrome, and CRRT. For example, prolonged MV time and tracheal re-intubation are well-recognized factors of pneumonia. The ECMO can cause SIRS and represent a poor prognosis [19]. The CRRT usually results from renal malperfusion in patents after ATAAD surgery [20].

The predisposed poor outcomes may effectively help us to investigate the definition of postoperative hyper-inflammation. That was the reason why cut-off points of PCT, CRP, IL-6, and SII were easily found. Based on these points, we defined postoperative hyper-inflammation. In univariable analysis, the definable hyper-inflammation is significantly associated with poor outcomes (OR 7.54, 95% CI 3.95–14.40, *P* < 0.001). And this definable hyper-inflammation is an independent risk factor of poor outcomes in multivariate analysis (Table 3). There are reasons to believe that our definition of hyper-inflammation is acceptable, useful and reliable. It will help clinicians to quickly and easily predict postoperative hyper-inflammation.

Based on our definition, we divided the study population into hyper-inflammation group and control group. After univariate and multivariable analysis, the pre-operative bowel ischemia, pre-operative limb ischemia, CPB > 180 min and DHCA > 40 min were independent risk factors for postoperative hyper-inflammation. Pre-operative bowel ischemia and pre-operative limb ischemia are unsurprised to be risk factors of hyper-inflammation. Because pre-operative limb and bowel ischemia had an inflammatory related pathophysiologic process of I/R injury during the period of ATAAD surgery. Previous studies also reported that limb and bowel ischemia, identified as high-risk factors of hyper-inflammation, could significantly increase in-hospital mortality [10, 11]. ATAAD surgery required prolonged CPB and DHCA, which allowed the release of huge inflammatory indicators in body, including PCT, CRP, and IL-6, and therefore they have been widely recognized as independent risk factors of hyper-inflammation [21, 22]. However, to our best knowledge, there were no large-sample sized studies to demonstrate which cut-off points of CPB and DHCA time could lead to hyper-inflammation in patients who underwent ATAAD surgery. The ATAAD,a rare and critical disease, is scattered far and wide across the country. It has to implement a multi-center cohort to collect the study population. Our hospital is a regional cardiovascular center, due to which many ATAAD cases accumulated. We built a cohort that enrolled 405 patients with ATAAD, and calculated that cut-off points of CPB and DHCA time were 180 min and 40 min, respectively. This is the first study to report the conclusion of time thresholds that CPB > 180 min and DHCA > 40 min were significantly associated with postoperative hyper-inflammation. In general, this work will more rigorously suggest surgeons should try their best to reduce the operative time, which might be the only way for any patient to have a positive prognosis.

### Study limitation

The current study had certain inherent limitations. Our study approach incorporates one center's experiences, which is a limitation of retrospective observational research. Retrospective observational studies are highly prone to bias. And other centers may have different results compared with our hospital. Our conclusions need these centers’ data to further test. Besides, due to limited research funding, we are unable to provide dynamic inflammatory indicators trends, which has an impact on our outcomes. However, according to previous findings in the literature, the inflammatory indexes on the first postoperative day were also highly suggestive of prognosis, and the conclusions of this study are indeed consistent with the previous literature [23]. Finally, our research population is the Chinese Han, which is noticeably different from the populations of the United States and Europe. The cut-off points may be different in the population from the US and Europe.

## Conclusion

According to your findings, the morbidity of hyper-inflammation was about 12.83% in patients after ATAAD surgery. It could lead to a poor prognosis. A combination of PCT > 2.18, CRP > 49.76, IL-6 > 301.88, and SII < 2509.96 could be used to define postoperative hyper-inflammation which increased mortality and morbidity in patients after ATAAD surgery. Moreover, pre-operative bowel ischemia, pre-operative limb ischemia, CPB > 180 min and DHCA > 40 min were independent risk factors for postoperative hyper-inflammation.

## Data Availability

The datasets generated and/or analysed during the current study are not publicly available due to patients did not signed consents about upload the data but are available from the corresponding author on reasonable request.

## Abbreviations

ATAAD: Acute type-A aortic dissection
CPB: Cardiopulmonary bypass
DHCA: Deep hypothermia circulatory arrest
PCT: Procalcitonin
IL-6: Interleukin-6
CRP: C-reactive protein
SII: Systemic immune-inflammation index
ACC: Aortic cross-clamp
POD1: First postoperative day
CABG: Coronary artery bypass grafting
MV: Mechanical ventilation
ECMO: Extracorporeal membrane oxygenation
CRRT: Continuous renal replacement therapy
